# Single cell RNA-seq data and bulk gene profiles reveal a novel signature of disease progression in multiple myeloma

**DOI:** 10.1186/s12935-021-02190-6

**Published:** 2021-09-25

**Authors:** Zhiyong Zeng, Junfang Lin, Kejie Zhang, Xizhe Guo, Xiaoqiang Zheng, Apeng Yang, Junmin Chen

**Affiliations:** 1 Department of Hematology, The First Affiliated Hospital of Fujian Medical University, Fuzhou, China; 2 Department of Hematology, Zhongshan Hospital Xiamen University, Xiamen, China; 3 Department of Hematology, The Second Affiliated Hospital of Fujian Medical University, Quanzhou, China

**Keywords:** Single cell RNA-seq, Bulk gene profiles, Novel signature, Disease progression, Multiple myeloma

## Abstract

**Background:**

The development of multiple myeloma (MM) is considered to involve a multistep transformation process, but the role of cytogenetic abnormalities and molecular alterations in determining the cell fate of multiple myeloma (MM) remains unclear. Here, we have analyzed single cell RNA-seq data and bulk gene profiles to reveal a novel signature associated with MM development.

**Methods:**

The scRNA-seq data from GSE118900 was used to profile the transcriptomes of cells from MM patients at different stages. Pseudotemporal ordering of the single cells was performed using Monocle package to feature distinct transcriptomic states of the developing MM cells. The bulk microarray profiles from GSE24080 and GSE9782 were applied to identify a signature associated with MM development.

**Results:**

The 597 cells were divided into 7 clusters according to different risk levels. They were initiated mainly from monoclonal gammopathy of undetermined significance (MGUS), newly diagnosed MM (NDMM), or relapsed and/or refractory myeloma (RRMM) with cytogenetically favorable t(11;14), moved towards the cells from smoldering MM (SMM) or NDMM without t(11;14) or t(4;14), and then finally to cells from SMM or RRMM with t(4;14). Based on the markers identified in the late stage, the bulk data was used to develop a 20-gene signature stratifying patients into high and low-risk groups (GSE24080: HR = 3.759, 95% CI 2.746–5.145; GSE9782: HR = 2.612, 95% CI 1.894–3.603), which was better than the previously published gene signatures (EMC92, UAMS70, and UAMS17) and International Staging System. This signature also succeeded in predicting the clinical outcome of patients treated with bortezomib (HR = 2.884, 95% CI 1.994–4.172, *P* = 1.89e−8). The 20 genes were further verified by quantitative real-time polymerase chain reaction using samples obtained from the patients with MM.

**Conclusion:**

Our comprehensive analyses offered new insights in MM development, and established a 20-gene signature as an independent biomarker for MM.

**Supplementary Information:**

The online version contains supplementary material available at 10.1186/s12935-021-02190-6.

## Introduction

Multiple myeloma (MM) is a plasma cell malignancy characterized by a spectrum of monoclonal gammopathy of undetermined significance (MGUS), smoldering MM (SMM), and newly diagnosed MM (NDMM), and ultimately progresses to a relapsed or refractory multiple myeloma (RRMM) [1–3]. Despite several advancements in the therapeutic approaches, MM remains an incurable disease. The heterogeneity of MM is increasingly being recognized, as its survival period ranges from < 6 months to > 10 years [4]. The gaps between our understanding regarding the full spectrum of cellular heterogeneity and the distinct cell types that comprises of human MM cells hinders our ability to explore their roles in tumorigenesis and its progression.

Many studies have reported the influence of the presence of cytogenetic abnormalities and molecular alterations in disease progression, response to therapy, and prognosis during the occurrence and development of MM [5, 6]. However, their role in determining the fate of MM cells still remains unclear. So, it is imperative to investigate more specific expression profiles of each human MM cell class.

With the development of high-throughput gene detection technologies, single cell RNA sequencing (scRNA-seq) assists in exploring cellular heterogeneity on a single cell level and reconstructs lineage hierarchies. Although several previous studies [6–8] have performed scRNA-seq on human MM cells, no clear discussion has been done on the relationship between cytogenetic abnormalities and MM cells differentiation trajectory. Hence, in the present study, a comprehensive analysis of scRNA-seq data and bulk gene expression profiles was performed to reveal the development map of human MM cells, and develop a novel gene signature in order to accurately predict the prognosis of MM.

## Methods

### Source and analysis of scRNA-seq data

The scRNA-seq data of MM cells was obtained from the GSE118900 dataset [6]. The dataset included transcripts of 597 individual MM cells from 15 patients (including MGUS, SMM, NDMM, and RRMM) with or without cytogenetic abnormality. The normalized scRNA-seq data was read as counts in the matrix and was analyzed by the Seurat version 3.0.2 [9]. The cells with less number of genes detected (i.e., < 200 genes) were regarded as outliers and therefore are excluded from the downstream analyses. As the increase in mitochondrial genes might be related to those cells experiencing stress and cell death, cells with a percentage of mitochondrial genes of less than 5% were also included. Individual cells were then normalized by log-normalization with a scale factor of 10,000. Variable genes were selected by FindVariableFeatures function to perform principal component analysis (PCA). The jackstraw method was used to quantify the *P*-value distribution of the top 20 PCAs [9]. The statistically significant PCAs were chosen for t-distributed stochastic neighbor embedding (t-SNE) followed by K-means clustering. The Find All Markers function in the Seurat package was used to identify the specific markers for each cluster. Heatmap was performed to represent the scaled expression data of these marker genes. Normalized data were illustrated as feature plots or violin plots. The pseudotemporal ordering of single cells using Monocle R package was performed to reveal the dynamic changes in the transcriptome of developing MM cells [10].

### Functional enrichment analyses

The R package clusterProfiler were performed to analyze and visualize functional profiles, including Gene Ontology (GO), Kyoto Encyclopedia of Genes and Genomes (KEGG) pathway analyses [11]. The GO terms or KEGG pathways with adjusted *P* values of less than 0.05 were considered to be significant. The top 10 GO terms were visualized by GOplot R package [12].

### Identification of gene signature based on bulk gene expression datasets

The bulk microarray profiles and clinical characteristics were extracted from the GSE24080 dataset [13] (samples from patients with NDMM enrolled into total therapy of 2 and 3 trials) and GSE9782 dataset [14, 15] (samples from patients with RRMM enrolled into the APEX trial). The datasets were annotated using the platforms GPL570 and GPL96, respectively. If there was a single gene matching multiple probe sets, then its average expression was computed. The purpose of this analysis was to extract the previously unreported but meaningful information and provide new biological insights based on the results of scRNA-seq data. To establish a multi-gene signature for predicting the prognosis of MM patients, the markers identified by scRNA-seq analysis were used in univariate Cox proportional hazards model and the least absolute shrinkage and selection operator (LASSO) regression analysis was carried out by the R package glmnet [13]. Furthermore, we assessed the performance differences between our signature and the previously published prognostic gene signatures (such as EMC92, UAMS70 and UAMS17) [16, 17], a RNA sequencing-based signature (RNAseq_signature) recently developed from The Cancer Genome Atlas (TCGA) MM RNA sequencing dataset (MMRF-CoMMpass)[18], and International Staging System (ISS) [4]. The cut-off values of published signatures were used as reported in the previous studies [16, 17].

### Gene set enrichment analysis (GSEA)

GSEA is a powerful computational method to determine whether a set of priori genes have statistically significant consistency between the two biological states (e.g., high risk vs. low risk) [19, 20]. Gene sets ‘Hallmarks v6.2’ were obtained from the MSigDB. The R package clusterProfiler was applied for GSEA analysis.

### Patient samples

The bone marrow samples from 20 patients with MM, including 5 patients with t(4;14) and 15 patients without t(4;14), were collected from the Department of Hematology, The First Affiliated Hospital of Fujian Medical University. Primary plasma cells were isolated from bone marrow specimens using anti-CD138 MicroBeads (Miltenyi, Germany) and immediately frozen in −80 °C until the subsequent extraction of RNA. All procedures of samples acquirements have followed the tenets of the Declaration of Helsinki and are reviewed by the Ethics Committee of The First Affiliated Hospital of Fujian Medical University.

### Quantitative real-time polymerase chain reaction (qRT-PCR) analysis

According to the manufacturer’s protocol, TRIzol reagent (Invitrogen) was used to extract total RNA of enriched primary plasma cells (2–3*10^6^). Total RNA (20–30 μg) of each sample was reverse transcribed into complementary DNA using a reverse transcription kit (Takara Bio, Inc.). QRT-PCR was performed on the Thermofisher 7500 PCR machine (Applied Biosystems, USA) with hamQ Universal SYBR qPCR Master Mix (Vazyme Biotech Co.,Ltd). The thermocycling conditions were as follows: initial denaturation at 95 ℃ for 30 s, followed by 95 ℃ for 10 s, 62 ℃ for 30 s, for 40 cycles. All experiments were done in triplicate, and the results were normalized to the expression of β-actin. The specificity of amplification was verified by melting curve analysis. Additional file 1: Table S1 presents the primer sequences of the hub genes. The data from qRT-PCR were analyzed by the 2^−ΔΔCt^ method [21].

### Statistical analysis

Kaplan–Meier curves with log-rank testing were applied to investigate the differences in survival between different groups of MM patients. Hazard ratio (HR) with 95% confidence interval (CI) was calculated. The area under the curve (AUC) was calculated by receiver operating characteristic (ROC) curve. The data are presented as the means ± standard deviation (SD). Differences were analyzed using the Student's t-test. Statistics software SPSS 20.0 (IBM, Chicago, IL)) and R software version 3.6.0 were used for data analysis. A two-sided *P* < 0.05 indicated a significant difference.

## Results

### ScRNA-seq profiling demonstrated gene expression patterns during disease development of MM

**Fig. 1:**
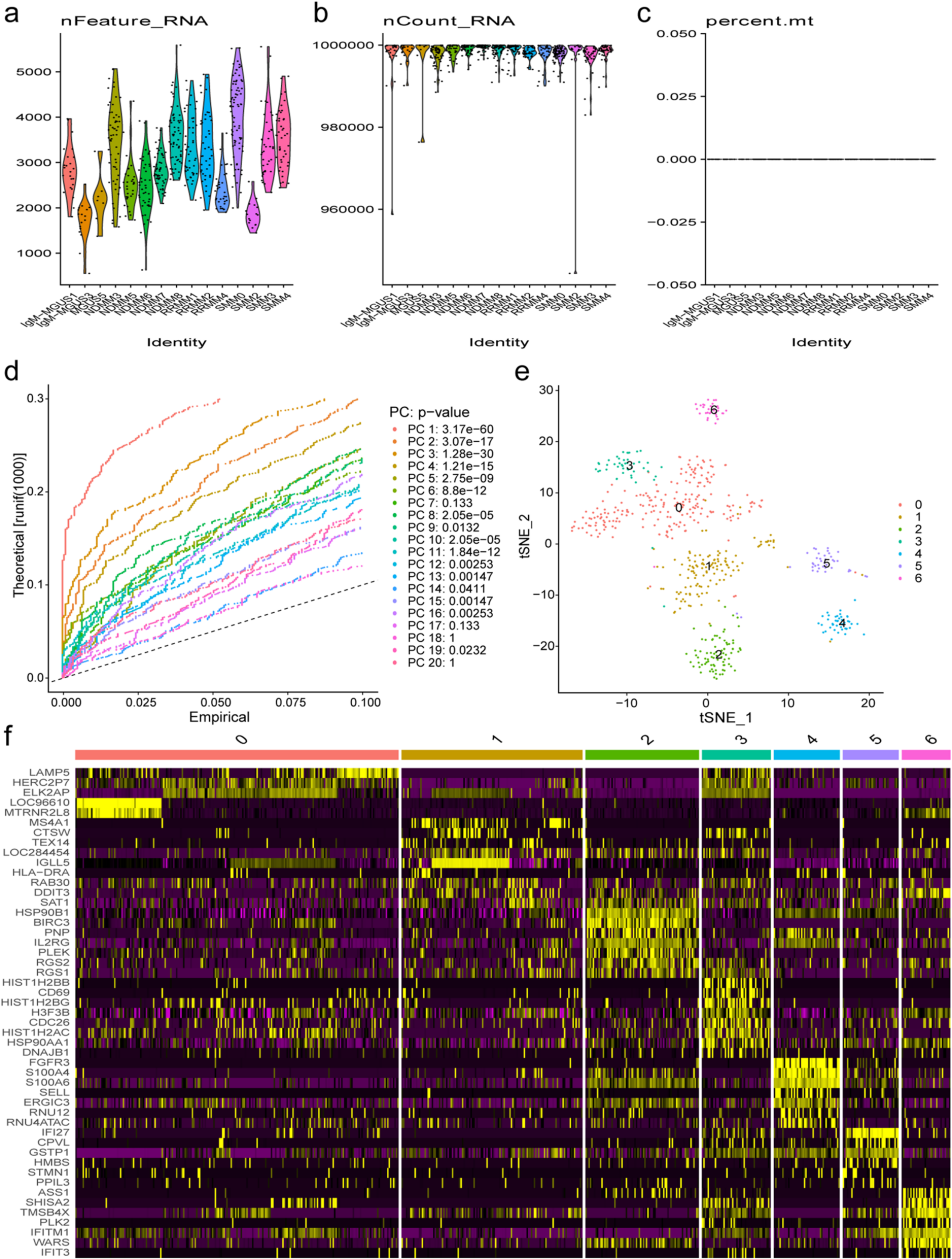
Single cell RNA-seq transcriptome profiling of human multiple myeloma (MM) cells from GSE118900 dataset. **a** Graph showing the distribution of the number of expressed genes in all single cells; **b** graph showing the total count distribution in all single cells; **c** graph showing the distribution of mitochondrial genome in all single cells; **d** the jackstraw method was used on the top 20 principal components of single cells collected from the MM cells; e the t-SNE projection of MM cells as determined by Seurat. Each dot corresponds to one individual cell; **f** Heatmap illustrated the expression patterns of the top ten markers in individual cells of each cluster by Seurat analysis. The columns correspond to the cells; and the rows correspond to the genes. Cells are grouped by clusters

From dataset GSE118900, a total of 23,398 transcripts in 597 individual cells isolated from 15 MM patients at different stages were downloaded and pre-processed for scRNA-sequencing analysis. The average expression value of 54 duplicate genes was calculated. After filtering the total number of genes that are expressed in a single cell and the percentage of mitochondrial reads (Fig. 1a–c), 597 cells with 16,568 expressed genes were identified. An unbiased PCA of the 597 cells was performed using highly variable genes to examine the global transcriptome patterns in the scRNA-seq data. The 16 statistically significant principal components in the PCA were reduced to two dimensions using t-SNE (Fig. 1d). The 597 cells were divided into 7 distinct clusters, each consisting of cells from MM patients at different stages (Fig. 1e). The expression patterns of markers in individual cells of each cluster were presented in Fig. 1f. A summary of clinical information, fluorescence in situ hybridization (FISH) results and clusters were shown in Table 1.

**Table 1:** Summary of patient characteristics and clusters identified by t-distributed stochastic neighbor embedding (t-SNE)

Sample IDs	Total number of cells analyzed	Cluster 0	Cluster 1	Cluster 2	Cluster 3	Cluster 4	Cluster 5	Cluster 6	Cytogenetic abnormality
IgM MGUS1	24	1(4%)	21(88%)	1(4%)	0	0	1(4%)	0	Not tested
IgM MGUS3	17	0	17(100%)	0	0	0	0	0	Not tested
MGUS5	7	0	6(86%)	0	0	0	1(14%)	0	Normal
SMM0	77	0	0	76(99%)	0	0	0	1(1%)	t(4;14), gain 1q21, del 13q
SMM2	16	1(6%)	15(94%)	0	0	0	0	0	t(14;20), monosomy 13
SMM3	39	36(92%)	3(8%)	0	0	0	0	0	Trisomy 7,9,11 and 15
SMM4	44	43(98%)	1(2%)	0	0	0	0	0	Trisomy 3, 7,9,11 14, & 15
NDMM3	59	10(17%)	2(3%)	0	47(80%)	0	0	0	Trisomy 3,7,& 11, trisomy/tetrasomy 9 & 15
NDMM5	32	28(88%)	4(13%)	0	0	0	0	0	Trisomy 7,9,11, &14, trisomy/tetrasomy 3 & 15, del 13q
NDMM6	47	46(98%)	1(2%)	0	0	0	0	0	Trisomy 3, 9, 11, &15
NDMM7	54	0	54(100%)	0	0	0	0	0	t(11;14)
NDMM8	60	59(98%)	1(2%)	0	0	0	0	0	Trisomy 3, 8, 9, &14, trisomy/tetrasomy 7, tetrasomy 11, gain 1q21
RRMM1	46	0	0	0	0	46(100%)	0	0	t(4;14), monosomy 13, del 17p
RRMM2	42	1(2%)	2(5%)	2(5%)	0	0	37(88%)	0	t(4;14), trisomy 11 & 15, monosomy 9 & 13
RRMM4	33	0	0	0	0	0	0	33(100%)	t(11;14) and tetraploid
Total	597	225(38%)	127(21%)	79(13%)	84(14%)	46(8%)	2(0%)	34(6%)	

As suggested in Table 1, a majority of the MM cells from the 3 MGUS patients were clustered into cluster 1 (92%), but there were fewer cells in clusters 0, 2, or 5 (2%, 2% and 4%, respectively). most of cells from patients (RRMM1, RRMM2 and SMM0) with t(4;14) translocation were clustered into clusters 2, 4, and 5 (99%, 100% and 88%, respectively). All the cells from patients (NDMM7 and RRMM4) with t(11;14) translocation were clustered into clusters 1 and 6, respectively. All cells from the samples (SMM2, SMM3, SMM4, NDMM3, NDMM5, NDMM6, and NDMM8) were mainly distributed in clusters 0, 1, and 3.

### The development of human MM cells

**Fig. 2:**
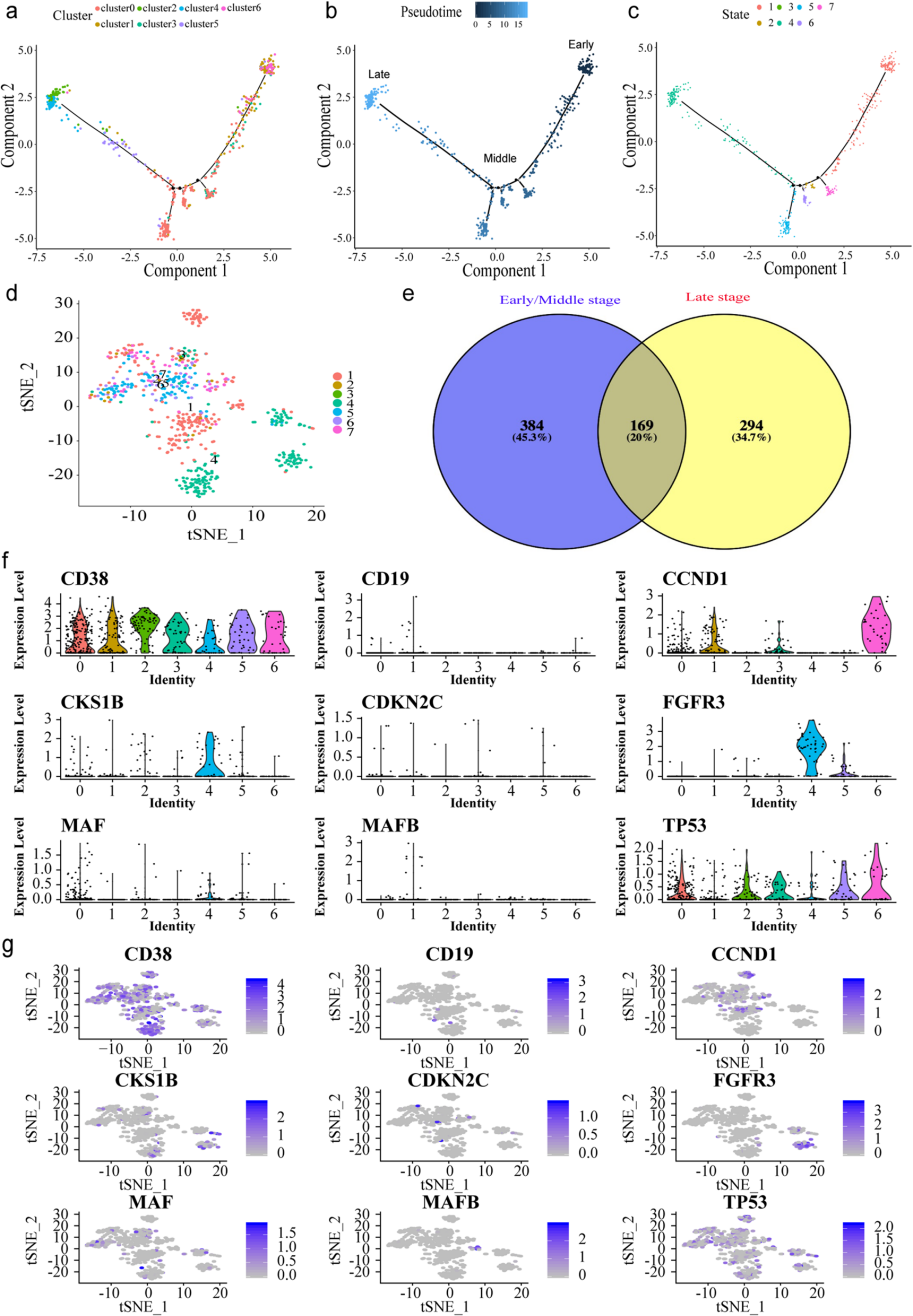
The development of human multiple myeloma (MM) cells. **a** Pseudotemporal trajectory of human MM cells assigned to clusters 0–6 using the Monocle 2 algorithm; **b** Pseudotime ordering of MM cells was shown in a gradient from dark to light blue; **c** Developmental pseudotime reflecting the cell state transition of MM cells; **d** t-SNE coloured by the different states; **e** Venn diagram of different marker genes between early/middle stage and late stage; **f** Violin plots of the expression patterns of representative genes of the MM cells; **g** The expression patterns of representative genes of the early and late MM cells mapped on t-distributed stochastic neighbor embedding (t-SNE) plot

To determine the relationship between these cell clusters and states, the differentiation trajectory and pseudo-time analysis was investigated using the Monocle2 R package based on the identified marker genes from each cluster (Fig. 2a–c). The MM cells could be divided into early, middle, and late stages. Based on the ordering of pseudotime, the MM cells appear to start principally from clusters 1 and 6 (state 1, most of the cells are from MGUS, NDMM or RRMM patients with cytogenetically favorable t(11;14) translocation), and moved towards clusters 0 and 3 (state 2, 3, 5, 6 and 7, all the cells from SMM or NDMM patients were without t(11;14) or t(4;14) translocation) and finally to clusters 5, 2, and 4 (state 4, most of the cells are from SMM or RRMM patients with cytogeneticly high risk t(4;14) translocation) (Fig. 2a–c). These results suggested that the MM cells were ordered in pseudotime that was consistent with the actual developmental stages. The t-SNE coloured by the states showed that cells at late stage (state 4) and early/middle stage (other states) can be distinguished clearly (Fig. 2d). Venn diagram indicated that there were 294 (34.7%) different marker genes between late stage and early/middle stage (Fig. 2e). To better understand the key genes that drive the ordinal construction of the manifold, representative genes of MM cells across the clusters were examined. As shown in Fig. 2f–g, the expression levels of CD38 were significantly increased and that of CD19 were markedly decreased, which in turn were consistent with the immunephenotype of malignant plasma cells. The common molecular cytogenetic abnormalities detected by FISH, including CKS1B, CDKN2C, TP53 and IgH partner genes (CCND1, FGFR3, MAF and MAFB), are related with the prognosis of patients.The expression levels of these genes were also detected. It is noteworthy that the MM cells during development showed a significantly increased expression levels of CCND1 in the early stage cells (cluster 1 and 6), but strikingly decreased expression levels in the late stage cells (cluster 2, 4, and 5). Furthermore, the expression level of FGFR3 was decreased in both early and middle stage cells (cluster 0, 1, 3 and 6), but was slightly or markedly increased in the late stage cells (cluster 2, 4 and 5).

### Functional enrichment analysis of markers of MM cells in the late stage

**Fig. 3:**
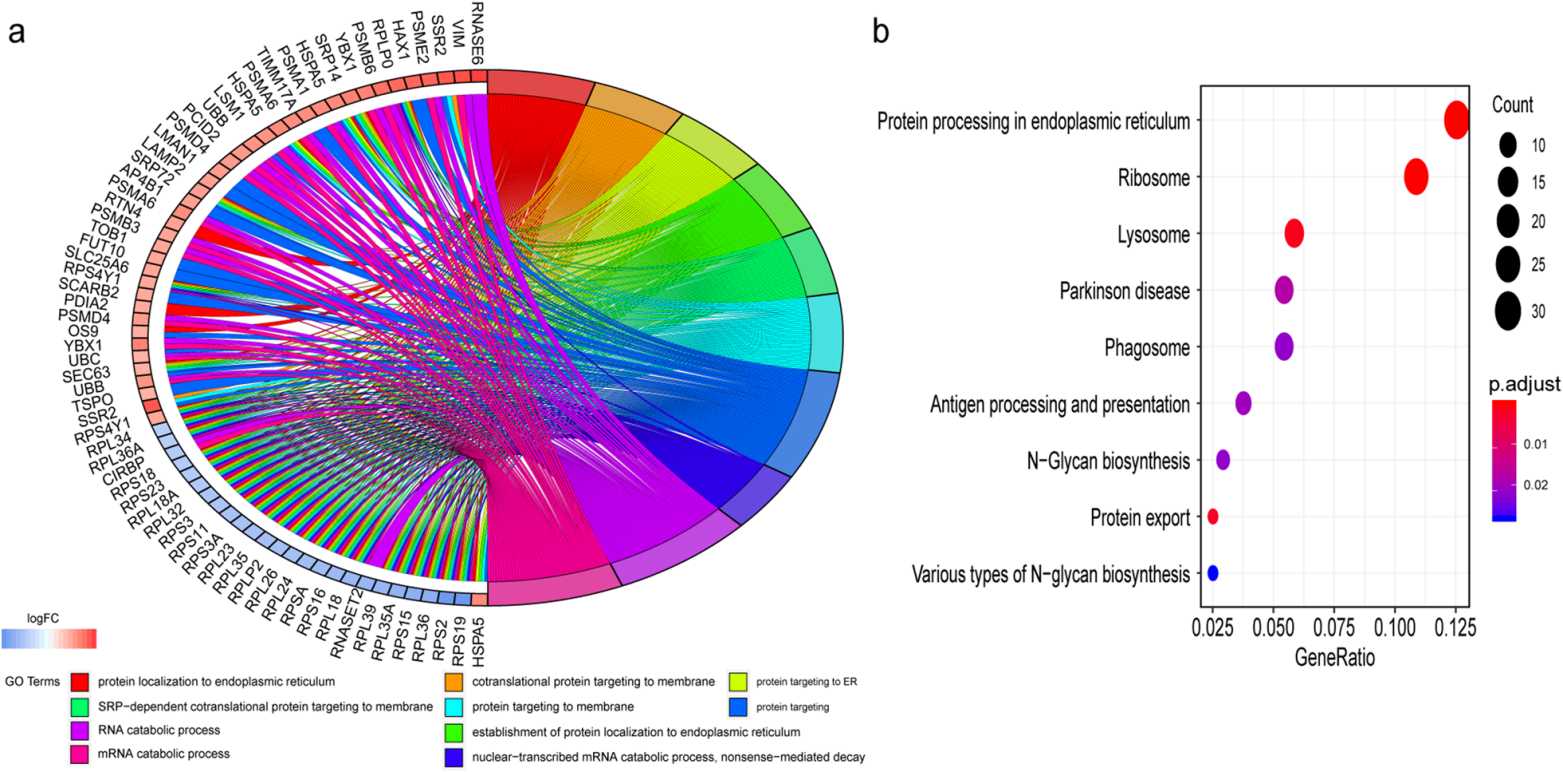
GO and KEGG pathway enrichment analysis of the markers in the late stage as identified by pseudotemporal analysis. **a** Chord plot depicting the relationship between the markers in the late state and the GO terms of biological process; **b** KEGG pathway analysis of the markers in the late state. GO, Gene Ontology; KEGG, Kyoto Encyclopedia of Genes and Genomes

To understand the functional insights into the markers of clusters 2, 4, and 5 in the late stage as identified by pseudotemporal analysis, an enrichment analysis using the clusterProfiler in R was performed. The top 10 GO biology processes (BP) and the top 9 KEGG terms are shown in Fig. 3. The results showed that the markers of clusters 2, 4, and 5 demonstrated significant enrichment in protein localization to endoplasmic reticulum, protein targeting, and RNA catabolic process. The significantly enriched KEGG pathways of the markers belonging to clusters 2, 4, and 5 showed protein processing in endoplasmic reticulum, ribosomes, lysosomes, and phagosomes. Taken together, these markers were mainly involved in protein processing, which might be associated with the characteristics of excessive accumulation of abnormal proteins in myeloma cells.

### Identification and validation of gene signature associated with MM progression

**Fig. 4:**
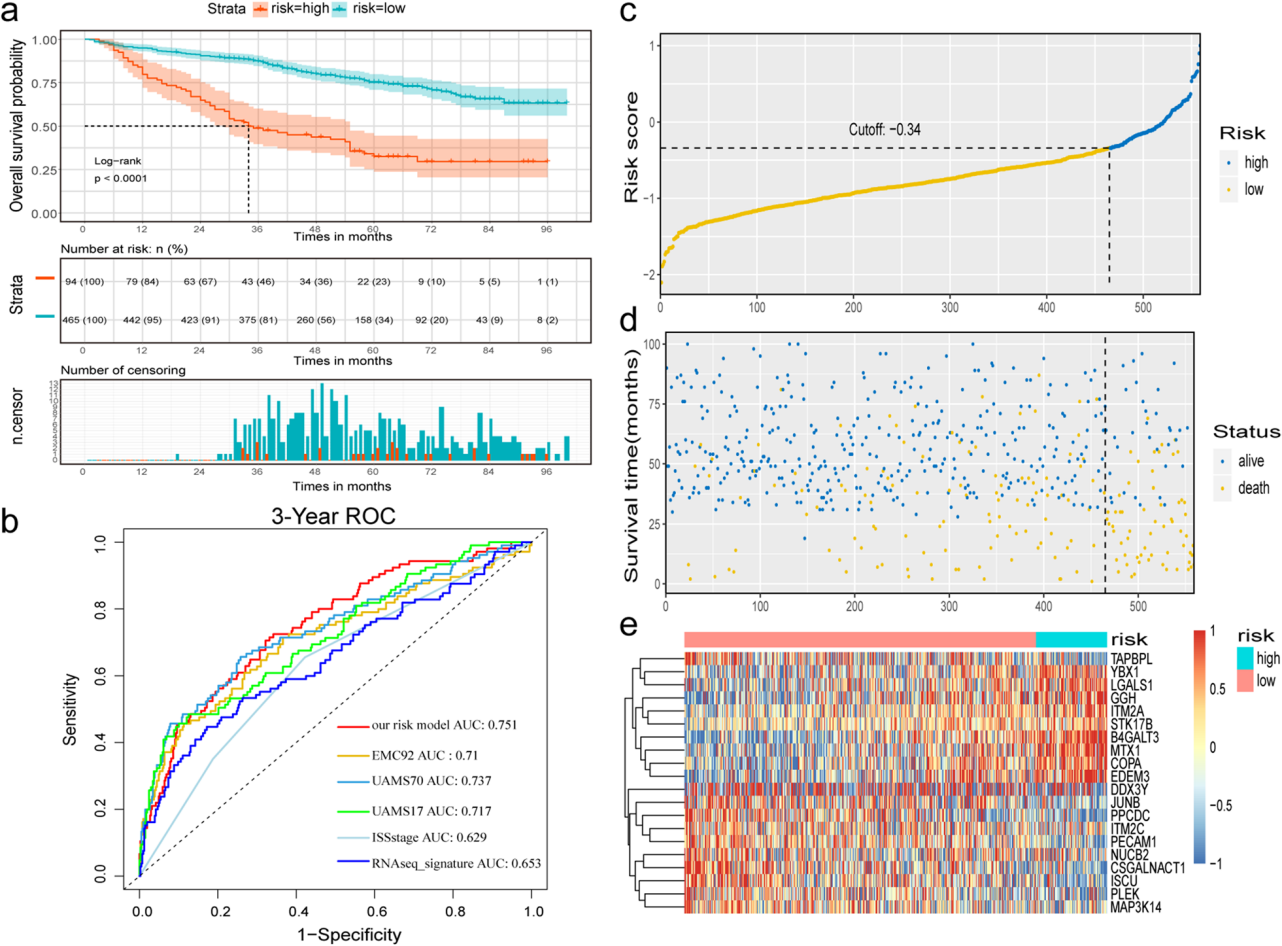
Prognostic evaluation of 20-gene signature in patients with newly diagnosed multiple myeloma from GSE24080 (n = 559). **a** Kaplan–Meier survival curves; **b** time-dependent operating characteristic curve analysis for survival prediction by using our risk model, EMC92, UAMS70, UAMS17, International Staging System stage (ISS), and RNAseq-based signature in the GSE24080 dataset; **c** the distribution of 20-gene risk scores; **d** patients’ survival status and time; **e** the heatmap of 20 genes expression in patients from the training set

GSE24080 was used as the training set to explore the prognostic value of 463 marker genes from MM cells in the late stage (clusters 2, 4, and 5) using univariate Cox regression analysis. All 90 genes that showed significant association with OS in MM patients were screened for LASSO regression. Eventually, the score formula comprised of 20 optimal genes that was developed by LASSO: risk score = 0.302*(expression level of B4GALT3) + 0.085*(expression level of EDEM3) + 0.083*(expression level of MTX1) + 0.061*(expression level of STK17B) + 0.047*(expression level of GGH) + 0.046*(expression level of YBX1) + 0.04*(expression level of ITM2A) + 0.035*(expression level of COPA) + 0.031*(expression level of LGALS1) − 0.002*(expression level of DDX3Y) − 0.01*(expression level of ITM2C) − 0.034*(expression level of MAP3K14) − 0.043*(expression level of TAPBPL) − 0.073*(expression level of JUNB) − 0.075*(expression level of CSGALNACT1) − 0.083*(expression level of PLEK) − 0.094*(expression level of NUCB2) − 0.107*(expression level of PECAM1) − 0.11*(expression level of ISCU) − 0.17*(expression level of PPCDC). The risk score of each sample in the training set and the optimal cut-off point of risk score (− 0.34) as determined by ROC analysis were calculated and was defined as the threshold. A total of 559 patients were divided into high-risk group (n = 94) and low-risk group (n = 465). Kaplan–Meier analysis demonstrated a significant difference in the survival rates between high-risk and low-risk groups (HR = 3.759 with 95% CI 2.746–5.145, Log-rank test *P* < 0.001, Fig. 4a). The median OS of high-risk group was 34.0 months, while that of low-risk group was not reached. Furthermore, the AUC obtained from the time-dependent ROC analysis for predicting OS was 0.751 at 3 years, demonstrating the performance of this signature predicting survival of MM patients better than those of the previously published signatures (EMC92, UAMS70, UAMS17, and ISS stages) (Fig. 4b). It was worth noting that AUC of our 20-gene signature based on microarray dataset was also higher than that of RNAseq_signature recently developed from RNA sequencing dataset MMRF-CoMMpass (AUC = 0.653) (Fig. 4b). The distribution of risk score, survival status and the heatmap of gene expression in patients from GSE24080 dataset were shown in Fig. 4c–e.

**Fig. 5:**
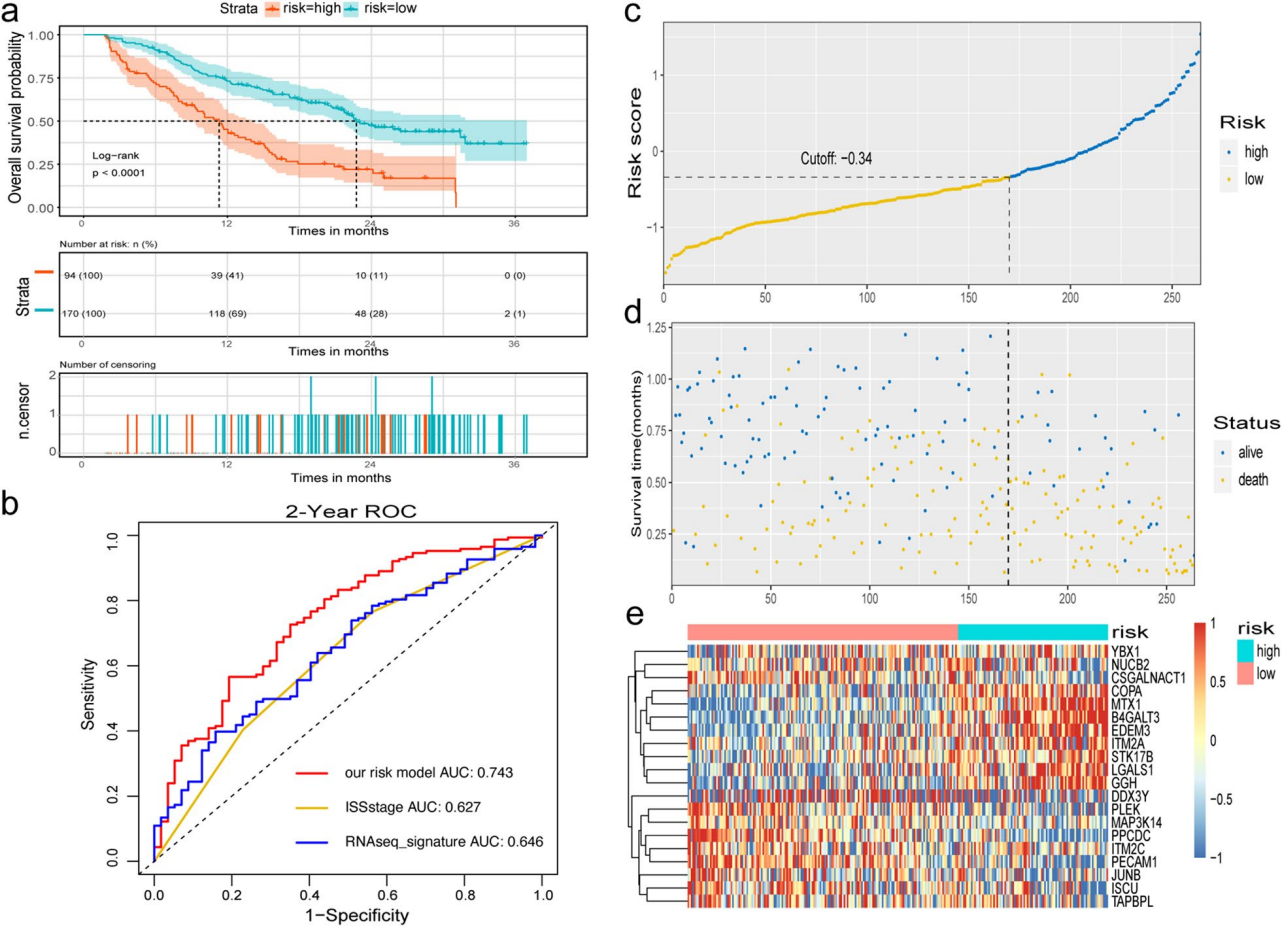
Validation of 20-gene signature in patients with relapsed MM obtained from the GSE9782 dataset (n = 264). **a** Kaplan–Meier survival curves; **b** Time-dependent operating characteristic analysis for survival prediction by our 20-gene risk model, International Staging System stage (ISS), and RNAseq-based signature in the GSE9782 set; **c** the distribution of 20-gene signature risk scores; **d** patients’ survival status and times; **e** the heatmap of 20 genes expression in patients from the training set

The power of the 20-gene prognostic model in predicting OS in patients with MM was validated as an independent dataset GSE9782. As shown in Fig. 5, patients with relapsed MM in GSE9782 dataset were divided into high-risk group (n = 94) and low-risk group (n = 170) by using the 20-gene signature as the same risk score formula and threshold, which was similar in the training set. Patients with high-risk exhibited poorer OS than those with low-risk (HR = 2.612 with 95% CI 1.894–3.603, *P* < 0.0001, Fig. 5a). The median OS of high-risk group was 11.3 months, while that of low-risk group was 22.8 months. As the platform GPL96 did not have all the probes of EMC92, UAMS70 or UAMS17, the performance of our 20-gene signature was unable to be compared with those of the published gene signatures. However, time-dependent ROC analysis still showed that the 20-gene signature achieved an AUC value of 0.743 at 2 years of OS, which was better than that of the ISS and RNAseq_signature(AUC = 0.627 and AUC = 0.646), (Fig. 5b). The distribution of risk score, survival status and the heatmap of gene expression in patients belonging to the dataset GSE9782 were shown in Fig. 5c–e.

### The 20-gene signature is independent of conventional clinical factors

The univariate and multivariate Cox regression analyses were performed to assess the independent predictive value of the 20-gene signature in the GSE24080 and GSE9782 datasets. In the training dataset GSE24080, the 20-gene signature and the clinical covariates of age, C-reactive protein (CRP), serum β2-microglobulin (β2M), serum creatinine (sCr), serum lactate dehydrogenase (LDH), serum albumin (ALB), hemoglobin (HGB) and bone marrow plasma cells (BMPC) demonstrated some prognostic value with the univariate Cox regression analysis (Table 2). The results revealed that HR calculated by the 20-gene risk fraction model (HR = 4.337, 95% CI 3.206–5.868, *P* = 1.83e−21) was higher than any single clinical covariate, demonstrating its higher prediction efficiency. After adjusting by sex and IgA_isotype, multivariate Cox regression analysis showed that the 20-gene prognostic model maintained a significant correlation with OS (HR = 3.532, 95% CI 2.625–4.87, *P* = 1.38e−14), indicating that the prognostic value of the 20-gene signature was an independent conventional prognostic factor for predicting patients with NDMM (Table 2). In the validation dataset GSE9782, both univariate and multivariate Cox regression analyses again showed 20-gene signature with highest HR as an independent prognostic factor in patients with relapsed MM (Table 2).

**Table 2:** Univariate and multivariate analyses for overall survival in patients with multiple myeloma obtained from the GSE24080 and GSE9782 datasets

Variables	Univariate analysis			Multivariable analysis		
	HR	95% CI	*P* value	HR	95% CI	*P* value
*Training set GSE24080*						
20-gene risk model (high/low)	4.337	3.206–5.868	1.83e−21^*^	3.532	2.625–4.87	1.38e−14^*^
Age (y)	1.024	1.007–1.041	5.21e−03^*^	1.009	0.992–1.026	0.315
Sex (male/female)	0.971	0.716–1.317	0.850	–	–	–
IgA_Isotype (Y/N)	1.093	0.777–1.537	0.609	–	–	–
Serum β2MG ≥ 3.5 mg/L (Y/N)	2.211	1.633–2.994	2.92e−07^*^	1.548	1.034–2.32	0.034^*^
CRP ≥ 8.0 mg/L (Y/N)	1.485	1.096–2.011	0.011^*^	1.14	0.822–1.58	0.433
sCr ≥ 2.0 mg/L (Y/N)	2.746	1.866–4.039	2.93e−07^*^	1.442	0.929–2.237	0.102
LDH > upper limit of normal (> 190 U/L) (Y/N)	2.32	1.716–3.137	4.57e−08^*^	1.502	1.078–2.093	0.016^*^
Serum ALB < 3.5 g/L (Y/N)	1.927	1.329–2.794	5.41e−04^*^	1.255	0.835–1.886	0.274
Hemoglobin < 100 g/L (Y/N)	1.625	1.191–2.216	2.17e−03^*^	0.982	0.693–1.391	0.916
Bone marrow plasma cells (%)	1.01	1.004–1.016	9.14e−04^*^	1	0.993–1.007	0.952
*Validation set GSE9782*						
20-gene risk model (high/low)	2.737	2.11–3.55	3.33e−14^*^	3.017	2.162–4.211	8.35e−11^*^
Serum β2M ≥ 3.5 mg/L (Y/N)	1.961	1.315–2.924	9.6e−04^*^	1.686	1.098–2.588	0.017^*^
CRP ≥ 8.0 mg/L (Y/N)	2.107	1.416–3.135	2.36e−04^*^	1.6	1.057–2.423	0.026^*^
Serum ALB < 3.5 g/L (Y/N)	1.798	1.298–2.49	4.17e−04^*^	1.618	1.058–2.476	0.026^*^

*HR* hazard ratio, *95%CI* 95% confidence interval, *β2MG* β_2_-microglobulin, *CRP* C-reactive protein, *sCr* serum creatinine, *LDH* lactate dehydrogenase, *ALB* albumin; *, statistically significant

### Clinical implications of genes associated with MM progression

**Fig. 6:**
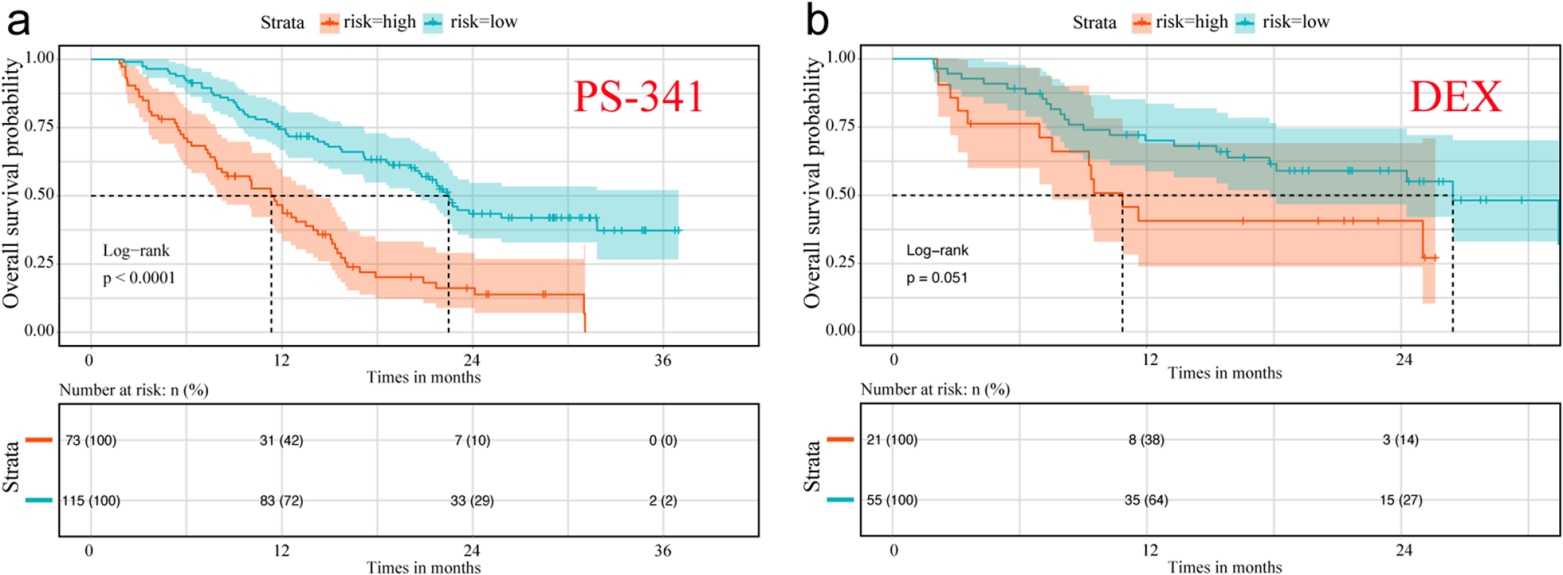
Kaplan–Meier analysis of the 20-gene prognostic signature in two main treatmentgroups in APEX trial. **a** Bortezomib (PS-341); **b** dexamethasone (DEX)

Using the APEX trial dataset (GSE9782) with available treatment information, this 20-gene signature succeeded in robustly discriminating between high- and low-risk patients in the bortezomib (PS-341) treatment group (HR = 2.884, 95% CI 1.994–4.172, *P* = 1.89e−8; Fig. 6a) but not in the dexamethasone (DEX) treatment group (HR = 1.956, 95% CI 0.983–3.891, *P* = 0.051; Fig. 6b).

### Validation of the hub genes

**Fig. 7:**
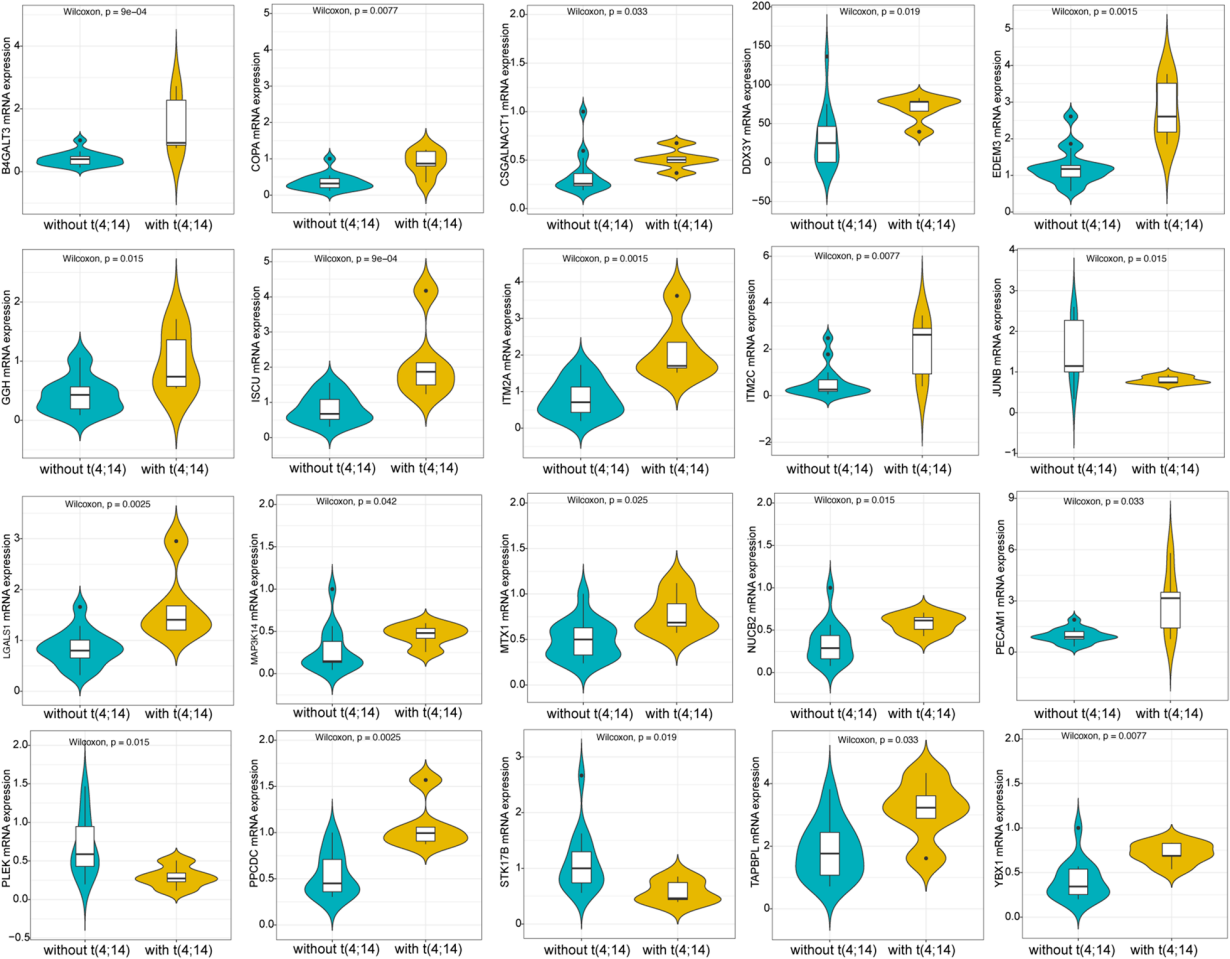
Quantitative real-time polymerase chain reaction (qRT-PCR) validation of the expression of 20 genes. The expression levels of genes (including B4GALT3, COPA, CSGALNACT1, DDX3Y, EDEM3, GGH, ISCU, ITM2A, ITM2C, JUNB, LGALS1, MAP3K14, MTX1, NUCB2, PECAM1, PLEK, PPCDC, STK17B, TAPBPL, and YBX1) in samples from multiple myeloma patients with t(4;14) and without t(4;14) were compared. ***P < 0.001; ** P < 0.01; *P < 0.05

QRT-PCR was used to further validate the expression levels of 20 hub genes (B4GALT3, EDEM3, MTX1, STK17B, GGH, YBX1, ITM2A, COPA, LGALS1, DDX3Y, ITM2C, MAP3K14, TAPBPL, JUNB, CSGALNACT1, PLEK, NUCB2, PECAM1, ISCU and PPCDC) in the primary plasma cells from 20 patients with MM, including 15 patients without t(4;14) and 5 patients with t(4;14). As shown in Fig. 7, except for the three genes (STK17B, JUNB, and PLEK), the expression levels of the other hub genes were up-regulated in the primary plasma cells from MM patients with t(4;14) compared to those without t(4;14), which was consistent with the markers at the late stage predictedby above mentioned bioinformatics analysis. Based on the median value (-0.19) of risk score calculated by 20 gene expression values of each sample, the 20 patients were divided into high-risk group (n = 10) and low-risk group (n = 10). As shown in Additional file 1: Table S2, patients in high-risk group were significantly correlated with elevated LDH, which has been considered as an indicator of poor prognosis in MM patients. These results confirmed that the 20-gene signature is a potential biomarker for MM.

### Pathway analysis by GSEA

**Fig. 8:**
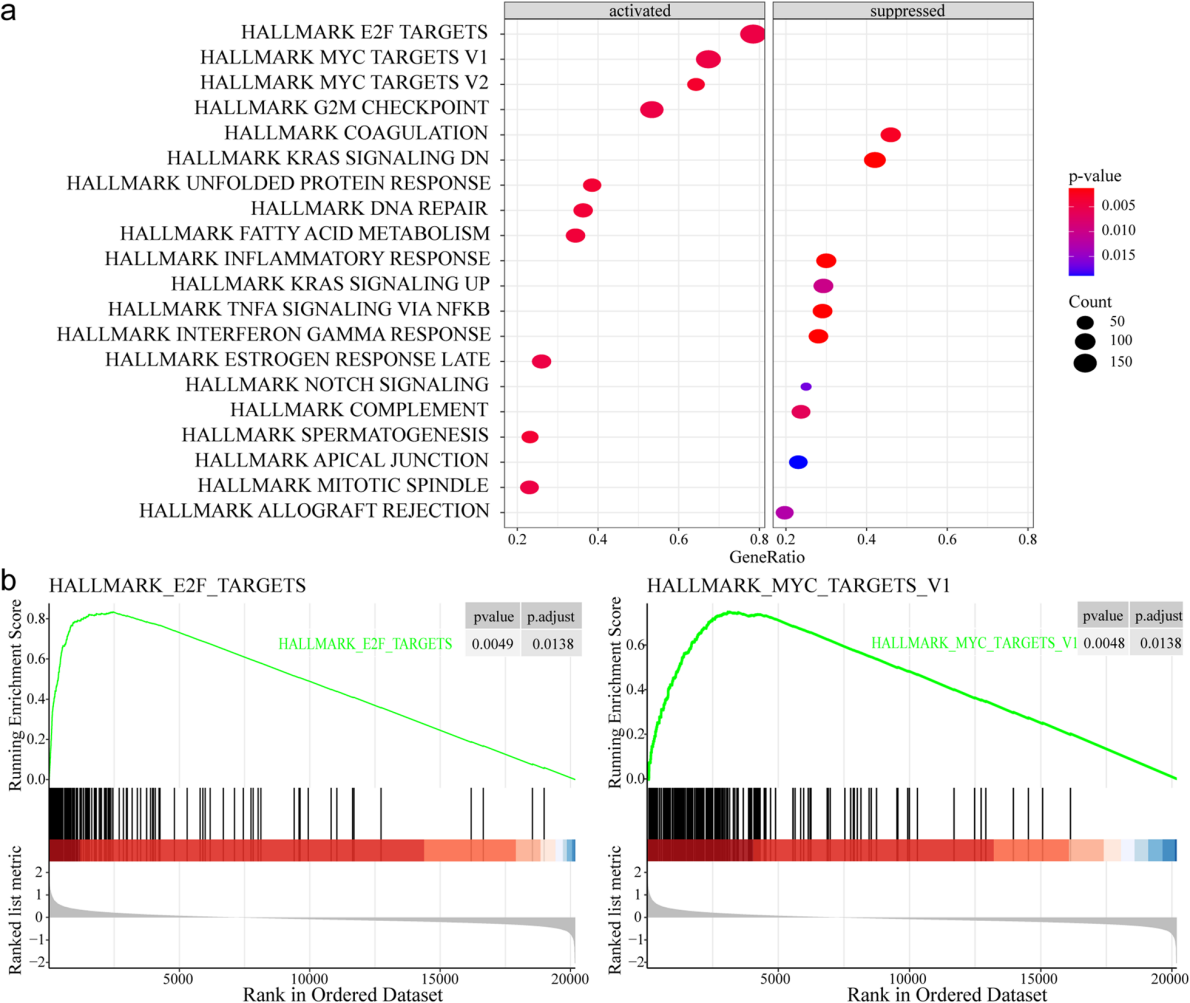
The hallmark gene sets were analyzed using Gene set enrichment analysis (GSEA) for high-risk and low-risk patients from GSE24080. **a** Activated and suppressed pathway analysis by using GSEA for the identified 20-gene signature. **b** Enrichment plots of GSEA revealing significant activated enrichments for E2F targets and MYC targets V1 gene sets

The GSEA was used to investigate the possible pathway in which the 20 genes might be involved. The expression profiles of high-risk and low-risk MM patients classified by 20-gene signature in the GSE24080 dataset were compared. The GSEA results revealed that the signaling pathways of hallmark E2F Targets, MYC Targets, G2M Checkpoint, Unfolded protein response, and DNA Repair showed significant activation in the high-risk group, while the hallmark KRAS signaling, inflammatory response, and TNFA signaling via NFKB were suppressed (Fig. 8).

### Discussion

The development of MM involves a multistep transformation process, in which genetic heterogeneity plays a crucial role [22–24]. There is a strong evidence that intraclonal heterogeneity exists in both MGUS, which is the earliest clinically recognizable stage of MM, and the later stages of the disease, including the high risk SMM and plasma cell leukemia [5, 25, 26]. A comprehensive analysis of scRNA-seq data and bulk gene expression profiles was performed to explore the role of genetic heterogeneity in MM disease progression. Although the previous studies used scRNA-seq to examine molecular heterogeneity in MM, none of them clearly defined the role of cytogenetic abnormalities in MM development. Jang et al. [6] have re-clustered cells from patients with MM into 4 sub-populations. Fan et al. [7] focused on copy number variation and loss of heterozygosity in individual cells in order to identify the major genetic subclones. Ledergor et al. [8] applied sc-RNA sequencing to study plasma cell heterogeneity in symptomatic and asymptomatic myeloma demonstrating high interindividual variability and identifying extensive subclonal structures. However, there is no in-depth analysis at the single cell level to explore the relationship between cytogenetics and MM cell differentiation trajectory.

To the best of our knowledge, this is the first study to explore the relationship between cytogenetic abnormalities and differentiation trajectory of MM cells. Based on unbiased clustering analysis and pseudotemporal reconstruction of differentiation trajectories, 597 cells from MM patients at different stages were re-clustered into 7 main groups according to different risk levels. The pseudotemporal reconstruction of differentiation trajectories yields a continuous lineage hierarchy. These were initiated mainly from the cells of MGUS, NDMM or RRMM patients with cytogenetically favorable t(11;14) translocation, moved towards the cells of SMM or NDMM patients without t(11;14) or t(4;14) translocation, and finally to the cells of SMM or RRMM patients with cytogenetically high risk t(4;14) translocation. It is noteworthy that the expression level of CCND1 showed a significant increase in the early stage cells when compared to the late stage cells, but FGFR3, an oncogene, showed a prominent increase in the late stage cells when compared to the early and middle stage cells. These results are consistent with the survival rate observed in MM patients with different cytogenetic abnormalities.

As t(4;14) is regarded as an important factor affecting the prognosis of patients with MM, the biological processes involved in the marker genes in the late stage cells were investigated. The enrichment results showed that the markers in the late stage were mainly involved in protein processing, indicating protein biosynthesis as a critical functional aspect that separates the late stage cells from the remaining cells.

To evaluate the prognostic value of markers in the late stage cells and to improve their prediction in MM, the markers related to OS of MM were screened from the public bulk gene expression profiles, and constructed a prognostic signature with significant genes. In this study, a novel 20-gene prognostic signature was developed to stratify patients into high- and low-risk groups with significantly different survival rate. Validation of the predictive value of the 20-gene signature was also successfully performed in an independent set. Within the same dataset GSE9782, the HR of the 20-gene signature (HR = 2.612 with 95% CI 1.894–3.603) was higher than that of the 44-gene signature (HR = 1.831 with 95% CI 1.33–2.522) developed by Jang [6], demonstrating the robustness and good reproducibility of 20-gene signature in MM. Further analysis revealed that the 20-gene risk model was independent of clinical prognostic variables. In addition, this signature succeeded in predicting the clinical outcome of patients treated with bortezomib. In particularly, the performance of the 20-gene signature in predicting the survival of patients was stronger than the previously published signatures (EMC92, UAMS70, and UAMS17) and the widely used ISS. As lack of enough information about revised international staging system (R-ISS),we were unable to compare our signature with R-ISS.It was noteworthy that AUC of our 20-gene signature based on microarray dataset was also higher than that of RNA sequencing-based signature recently developed from TCGA MM RNA sequencing datasetMMRF-CoMMpass. These results demonstrated that the 20-gene prognostic model could provide additional predictive information at the molecular level.

Validation of the expression levels of 20 hub genes by qRT-PCR confirmed that the 20-gene signature is a potential biomarker for MM.Furthermore, the GSEA was used to investigate the possible pathway involved in the 20 genes. The GSEA results showed that the signaling pathways of hallmark E2F Targets, MYC Targets, G2M Checkpoint, Unfolded protein response, and DNA Repair showed significant activation in the high-risk group, which were important in the development of MM. Thus, 20 genes might participate in the pathogenesis of MM by regulating these known biological processes and pathways.

Here are still some limitations in the present study. First of all, this prognostic signature is developed using retrospective analysis based on public datasets, thus, the results need to be further confirmed in prospective trails. Second, our research is based on data analysis combined with our own patient samples, but the small sample size, especially patients with t(4;14), is a limitation of the present study. Besides, our results are based on single cell sequencing and microarray, for transcriptome expression profile from RNA sequencing, its performance remains unknown.

In summary, the scRNA-seq data was used to cluster individual cells from 15 MM patients at different stages into 7 main clusters based on increasing risk levels in MM, and revealed the relationship between cytogenetic abnormalities and the differentiation trajectory of MM cells. Cells with cytogenetically favorable t(11;14) translocation showed association with early stage of MM, while cells with cytogenetically high risk t(4;14) were associated with the late stage of MM. Based on bulk gene expression profiles, an excellent and robust 20-gene signature was further developed, which in turn serves as an independent biomarker to predict survival of MM patients. Our results are considered to be important for understanding the pathological mechanisms and in designing the strategies for preventing MM progression.

## Supplementary Information

**Additional file 1: Table S1.** Primers used for the examined genes. **Table S2**. Clinical characteristics of 20 patients with multiple myeloma.

## Data Availability

The gene expression data and clinical data in this study can be found online at the Gene Expression Omnibus under accession numbers GSE118900, GSE24080 and GSE9782. The Cancer Genome Atlas (TCGA) MM RNA sequencing dataset (MMRF-CoMMpass) was also used in this study.

## Abbreviations

ALB: Albumin
95%CI: 95% Confidence interval
β2M: β2-Microglobulin
CRP: C-reactive protein
GO: Gene Ontology
GSEA: Gene set enrichment analysis
HR: Hazard ratio
KEGG: Kyoto Encyclopedia of Genes and Genomes
LDH: Lactate dehydrogenase
MGUS: Monoclonal gammopathy of undetermined significance
MM: Multiple myeloma
NDMM: Newly diagnosed multiple myeloma
PCA: Principal component analysis
sCr: Serum creatinine
scRNA-seq: Single cell RNA sequencing
SMM: Smoldering multiple myeloma
RRMM: Relapsed or refractory multiple myeloma
t-SNE: T-distributed stochastic neighbor embedding
