# Supplementary material for: Single cell RNA-seq data and bulk gene profiles reveal a novel signature of disease progression in multiple myeloma
Source: Cancer Cell Int. 2021 Sep 25;21:511. doi: 10.1186/s12935-021-02190-6 (PMC8465778; doi:10.1186/s12935-021-02190-6)
Supplement: Supplementary file 1 — Additional file 1: Table S1. Primers used for the examined genes. Table S2. Clinical characteristics of 20 patients with multiple myeloma. [file 12935_2021_2190_MOESM1_ESM.docx]

**Table S1.** Primers used for the examined genes

| Genes | Forward primer primer (5’-3’) | Reverse primer (5’-3’) |
| --- | --- | --- |
| β-actin | ACCGAGCGCGGCTACAG | CTTAATGTCACGCACGATTTCC |
| B4GALT3 | CGAGATCAGGGACCGACATTT | GATCGTTCTGGACAGTAGGGC |
| EDEM3 | CGAGCCCATGAGTAGGGAG | AAAGGCATGAGTTCATCAGCA |
| MTX1 | TGCTGACCTATGCCAGATTTACT | TGTGTGGAACTGAGATGACCT |
| STK17B | GCCTGTGTTTACCTGAGTTGG | TGTCCCCGAGAGGGTATATGC |
| GGH | GGCTGGATCTTACAGAGAAAGAC | ACTCTCCACTAATCAGCAGTGA |
| YBX1 | GGGGACAAGAAGGTCATCGC | CGAAGGTACTTCCTGGGGTTA |
| ITM2A | ATCCTGCAAATTCCCTTCGTG | CAGGTAAGCAGTCATTCCCTTT |
| COPA | TCAGCTTTCACCCCAAAAGAC | CACATCCGATAGTCCCATAACTG |
| LGALS1 | TCGCCAGCAACCTGAATCTC | GCACGAAGCTCTTAGCGTCA |
| DDX3Y | TGGACGGAGTGACTATGATGG | TAATCCCCGTGTTTCCTCCAG |
| ITM2C | GTGGTGTGCTGTATGAGGACT | CGTAGTTCTCGTCGAGGTAGAT |
| MAP3K14 | CGGAAAGTGGGAGATCCTGAA | GGGCGATGATAGAGATGGCAG |
| TAPBPL | CTGCCTGGCTCTATCTGGAG | CCTTGGAAATCGGTGAAGTCC |
| JUNB | ACGACTCATACACAGCTACGG | GCTCGGTTTCAGGAGTTTGTAGT |
| CSGALNACT1 | GCTGTGCTATCTCTGTCCTGT | CTTCAGGCTGCTCACGTAGTT |
| PLEK | AAGAAGGGGAGCGTGTTCAAT | TCAGCGGGATCATTCCTTTGG |
| NUCB2 | ATGAGGTGGAGGACCATCCTG | TGGTTCTATCTTCGCACTTTCC |
| PECAM1 | AACAGTGTTGACATGAAGAGCC | TGTAAAACAGCACGTCATCCTT |
| ISCU | GGGTCCCTTGACAAGACATCT | CCTTTCACCCATTCAGTGGCTA |
| PPCDC | TGCCTCTTCTGGTGTCAAAGC | TGTTTGGCTCTCTCAGTTGTGA |

**Table S2. Clinical characteristics of 20 patients with multiple myeloma**

| **Characteristics** | **Low risk (n=10)** | **High risk (n=10)** | **P value** |
| --- | --- | --- | --- |
| Age | 63.5±9.79 | 61.3±9.82 | 0.677 |
| Female sex, no. (%) | 2(20) | 4(40) | 0.314 |
| Hemoglobin (g/L) | 87.6±17.7 | 98.3±28.45 | 0.273 |
| Creatinine (μmol/L) | 178.48±224.9 | 147.87±233.01 | 0.112 |
| Serum calcium (mmol/L) | 2.15±0.26 | 2.21±0.24 | 0.427 |
| Albumin (g/L) | 34.4±6.08 | 34.83±7.38 | 0.762 |
| Lactate dehydrogenase (U/L) | 164.98±43.81 | 246.6±80.78 | 0.013 |
| β2-microglobulin (mg/L) | 6.73±4.34 | 5.2±2.68 | 0.45 |
| C-reactive protein (mg/L) | 12.1±18.38 | 32.13±43.31 | 0.096 |
| International Staging System, no. (%) |  |  | 0.097 |
| I | 0(0) | 2(20) |  |
| II | 3(30) | 4(40) |  |
| III | 7(70) | 4(40) |  |
